# Lithium niobate thin film electro-optic modulator

**DOI:** 10.1515/nanoph-2023-0865

**Published:** 2024-02-15

**Authors:** Jikun Liu, Lun Qu, Wei Wu, Chunyan Jin, Zhihao Chen, Zhidong Gu, Weiye Liu, Chenxiong Wang, Dahuai Zheng, Hongde Liu, Wei Cai, Mengxin Ren, Jingjun Xu

**Affiliations:** The Key Laboratory of Weak-Light Nonlinear Photonics, Ministry of Education, School of Physics and TEDA Applied Physics Institute, Nankai University, Tianjin 300071, People’s Republic of China; Collaborative Innovation Center of Extreme Optics, Shanxi University, Taiyuan, Shanxi 030006, People’s Republic of China

**Keywords:** lithium niobate film, Pockels effect, Fabry–Pérot resonance, electro-optic modulator

## Abstract

The linear electro-optic effect offers a valuable means to control light properties via an external electric field. Lithium niobate (LN), with its high electro-optic coefficients and broad optical transparency ranges, stands out as a prominent material for efficient electro-optic modulators. The recent advent of lithium niobate-on-insulator (LNOI) wafers has sparked renewed interest in LN for compact photonic devices. In this study, we present an electro-optic modulator utilizing a thin LN film sandwiched between top and bottom gold (Au) film electrodes, forming a Fabry–Pérot (F–P) resonator. This resonator exhibits spectral resonance shifts under an applied electric field, enabling efficient modulation of reflected light strength. The modulator achieved a 2.3 % modulation amplitude under ±10 V alternating voltage. Our approach not only presents a simpler fabrication process but also offers larger modulation amplitudes compared to previously reported metasurface based LN electro-optic modulators. Our results open up new opportunities for compact electro-optic modulators with applications in beam steering devices, dynamic holograms, and spatial light modulators, and more.

## 1 Introduction

The linear electro-optic effect (also called the Pockels effect) is a phenomenon in which the refractive index of a material changes linearly in response to an applied electric field [1]. This effect provides a means to control the properties of light as it traverses the material, presenting diverse applications in optical communication networks, sensing systems, and quantum information processing. Various non-centrosymmetric materials exhibits the Pockels effect, including lithium niobate (LN) [2], [3], [4], electro-optic polymers [5], [6], aluminum nitride [7], [8], and lead zirconate titanate [9], [10]. Among the forefront candidates in this domain, the LN’s unique combination of high electro-optic coefficients, wide optical transparency range (0.35–4.5 μm), and excellent chemical and mechanical stability positions it as a prime material for developing efficient electro-optic modulators [11], [12].

The recent emergence of lithium-niobate-on-insulator (LNOI) wafers has reignited significant interest in the LN within the field of integrated photonics [13], [14], [15], [16], [17], [18], [19], [20], [21], [22]. This heightened attention is credited to its remarkable capability to confine optical modes within nanometer dimensions, owing to the high refractive index contrast between the LN film and the ambient media [23], [24]. The LNOI electro-optic modulators have been a subject under continuous research in recent years. Notable examples include on-chip electro-optic modulators based on diverse LNOI microstructures, such as Mach–Zehnder interferometric waveguides [25], [26], photonic crystals [27], micro-rings [28], [29] or micro-disks [30], [31]. In these frameworks, the light propagation is confined within the plane of the electro-optic modulators, aiming for the application of integrated photonic circuits.

Moreover, there is a tremendous interest in realizing electro-optic manipulation of light waves in the free space. This capability is desirable for applications in a diverse range of fields, impacting technologies from displays [32], [33] and optical steering [34], [35], [36] to advanced light detection and ranging (LIDAR) technologies [37], [38]. In this context, electro-optic modulators based on monolithic LN metasurfaces [39], [40], or the hybrid integration of LN film with metallic metasurfaces [41], [42], [43], [44] have been demonstrated. Their underlying principle relies on the spectral resonance shifts due to the refractive index changes by the electro-optic effects. Noteworthily, the planar thin LN film, devoid of micro-structuring, can function as a resonator as well, exhibiting a series of Fabry–Pérot (F–P) resonances in the spectrum which are also tunable by the electro-optic effects. Consequently, this thin film offers a feasible framework for electro-optic modulation, with an added advantage of simpler fabrication compared to the metasurfaces.

In this work, we demonstrate an electro-optic modulator featuring a thin LN layer sandwiched between top and bottom gold (Au) film electrodes. The structure functions as a F–P resonator, presenting resonance dips in the reflection spectrum. To capitalize on the LN’s largest electro-optic coefficient, we applied the modulating electric field along the optic axis of the LN and introduced obliquely incident light polarized within the incident plane. The modulation amplitude of about 2.3 % at a modulating voltage of ±10 V was achieved, demonstrating better advantages over current work on LN metasurface-based electro-optic modulators due to our simple fabrication process and larger modulation amplitude.

## 2 Results

**Figure 1:**
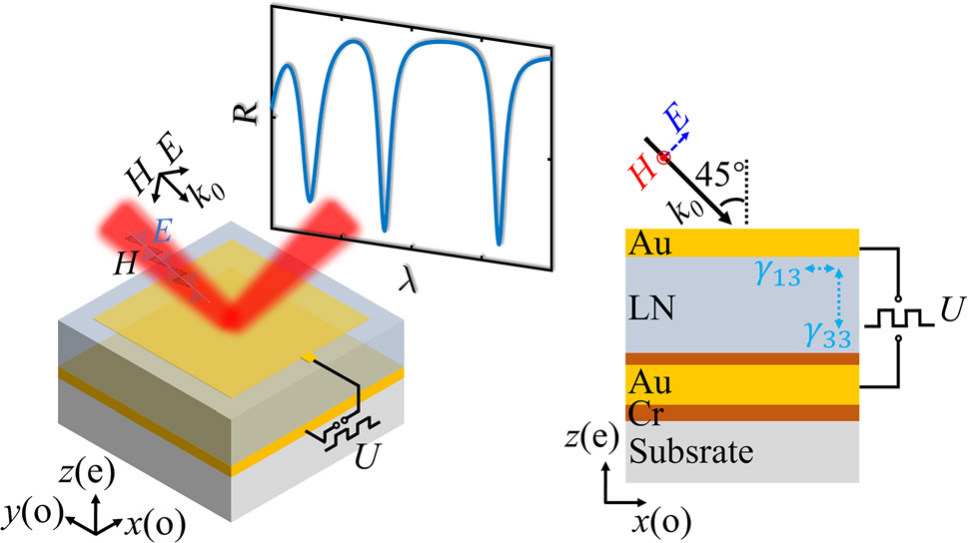
Schematics of the designed electro-optic modulator. The modulator comprises a thin Au film serving as a top electrode, deposited on a *z*-cut LN film. This LN film is attached to a continuous thin Au back electrode via a thin chromium adhesive layer. The entire structure forms a F–P cavity, exhibiting a series of resonance in the reflection spectrum. The right panel presents the cross-section of the device. External modulating voltage (*U*) is applied between the top and bottom electrodes, generating a modulating electric field along the optic axis (*z*-axis) of the LN. A light beam is incident at a 45° angle, with its electric field aligned within the incident plane (*p*-polarization), which includes both *e*- and *o*-polarization components. This configuration involves contributions from electro-optic coefficients of both *γ*_33_ and *γ*_13_.

Figure 1 illustrates the proposed structure, which consists of a 20 nm thick semitransparent Au film deposited on a commercial LNOI wafer (purchased from NANOLN Co. Ltd.). The LNOI wafer consists of a 707 nm thick *z*-cut LN film connected to a 100 nm Au film back-reflector through a 10 nm chromium (Cr) adhesive layer, which is further affixed onto a 500 μm thick LN substrate via a 30 nm Cr film and a 2 μm silica buffer layer, as shown by the cross-section on the right. The upper and lower Au films, combined with the sandwiched LN film, form an F–P resonator, which exhibits a series of resonance dips in reflection spectrum. These Au films also function as electrodes, creating an electric field along the *z*-direction (*E*_*z*_) within the LN thin film upon applying electrical voltage *U*. This electric field alters the refractive index according to the Pockels effect of the LN, leading to shifts in spectral resonances and changes in reflection magnitude at specific wavelengths.

The ellipsoid relation of the refractive index of LN under the voltage *U* can be described in a Cartesian coordinate system as:

(1) $$\begin{array}{ll} & \left(\frac{1}{n_{o}^{2}}+\gamma_{13}\frac{U}{d}\right)x^{2}+\left(\frac{1}{n_{o}^{2}}+\gamma_{13}\frac{U}{d}\right)y^{2}+\left(\frac{1}{n_{e}^{2}}+\gamma_{33}\frac{U}{d}\right)z^{2}=1, \end{array}$$

where *n*_*o*_ and *n*_*e*_ are the ordinary (*o*) and extraordinary (*e*) refractive indices of the LN without *U*, respectively. *d* is the LN thickness. *γ*_33_, with a value of 31.45 pm/V, corresponds to the *z* − *z* component of the Pockels tensor. This coefficient changes *n*_*e*_ under (*U*) through the relation [45]:

(2) $$|\Delta n_{e}|=\frac{1}{2}n_{e}^{3}\gamma_{33}\frac{U}{d}.$$

Similarly, *γ*_13_, measuring as 10.12 pm/V, modifies *n*_*o*_. Due to the larger value of *γ*_33_ compared with *γ*_13_, the modulation of *n*_*e*_ is more than three times greater than that of the *n*_*o*_ under the same voltage.

**Figure 2:**
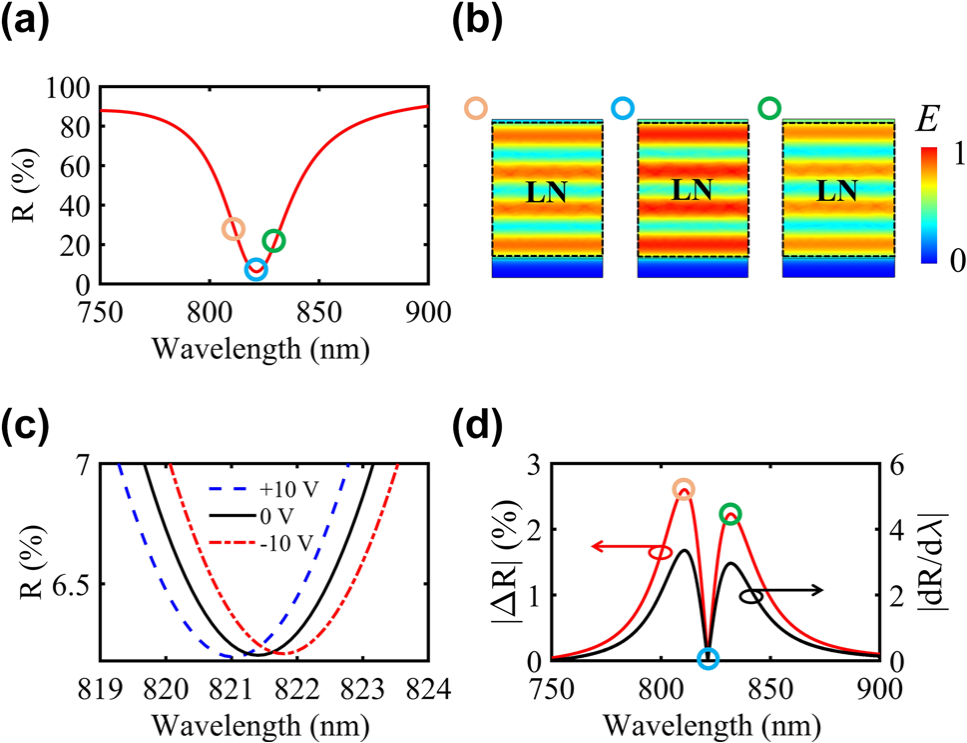
Simulated performance of the electro-optic modulator. (a) Simulated reflection spectrum (*R*) of the modulator under *p*-polarized light incident at 45°. (b) Optical electric-field distributions in the LN film at 810.7, 821.4, and 831.8 nm, respectively, which are away from and on a F–P resonance. (c) Spectral resonances at close range under various external modulating voltages. Dashed blue line: +10 V, solid black line: unmodulated spectrum, dashed red line: −10 V. (d) Reflectance changes (|Δ*R*|, left axis) between the −10 V and +10 V conditions. The absolute slope (|d*R*/d*λ*|, right axis) of the reflection spectrum is given by a black line.

We utilized *p*-polarized light with oblique incidence at 45°, where the electric field aligns within the incident plane and comprises both *e*- and *o*-polarization components. This configuration ensures that the larger electro-optic coefficient *γ*_33_ contributes to the modulation effect, surpassing the capabilities of *s*-polarized light, which interact solely with *γ*_13_. We used the finite element method (COMSOL Multiphysics software) to simulate the performance of the modulator. The material properties of Au and Cr are adopted from the Ref. [46] and [47], and the refractive indices of the LN were measured using an ellipsometer (Accurion EP4) [48]. In Figure 2(a), the reflection spectrum is presented, revealing a resonance dip at 821.4 nm. Figure 2(b) illustrates the optical electric-field distributions within the LN film at different wavelengths around the resonance, all exhibiting a standing wave pattern, indicative of the F–P nature of the resonance. This resonance efficiently increases the photon lifetime inside the LN, enhancing the strength of the light–matter interaction. The electro-optic effect, altering the refractive index of LN, effectively varies the optical length of the LN cavity, leading to a spectral shift. Figure 2(c) displays the calculated reflection spectra for different *U* values of +10 (blue), 0 (black), and −10 V (red), respectively. A spectral shift of 0.8 nm is achieved between −10 and +10 V, resulting in an approximate tuning sensitivity of Δ*λ*/Δ*V* = 0.04 nm/V. To further evaluate the modulator’s performance, the modulation amplitude is defined as the absolute difference between reflection (|Δ*R*|) under different *U* values. Figure 2(d) presents the |Δ*R*| (red curve) by subtracting the −10 V spectrum in Figure 2(c) from the +10 V one. The |Δ*R*| curve exhibits an M-shape, whose peaks are not at the reflection resonance wavelength of 821.4 nm, but rather at 810.7 and 831.8 nm, coinciding with positions of the highest slope of the reflection spectrum (black curve in Figure 2(d)). This is consistent with the fact that the sharp resonance, with the steep spectral slope, is beneficial to enhance the electro-optic modulation [49], [50].

**Figure 3:**
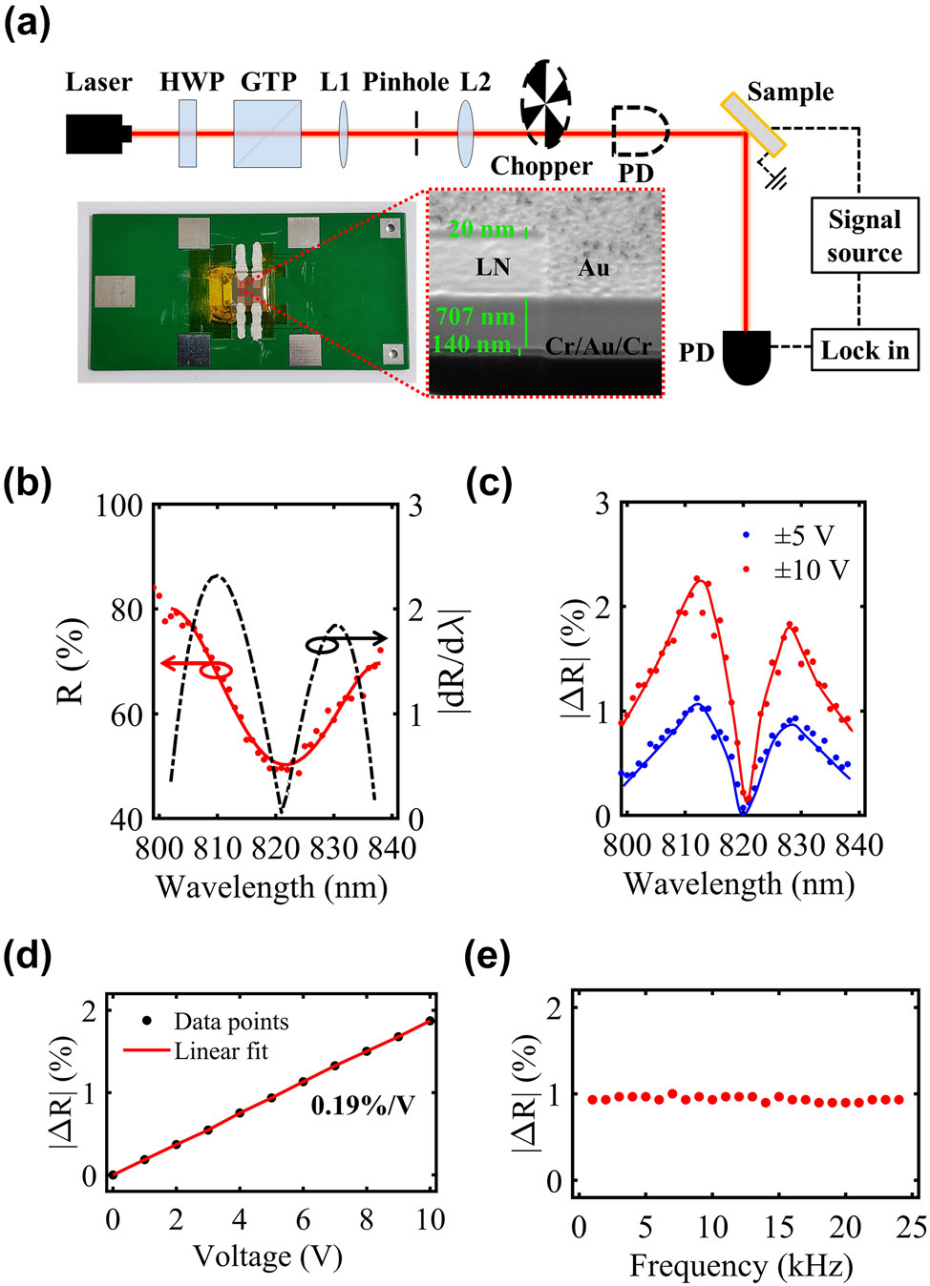
Experimental characterization of the electro-optic modulator. (a) Experimental setup includes a tunable laser, a power attenuator [comprising a half-wave plate (HWP) and a Glan-Taylor prism (GTP)], a beam collimator (consisting of a lens pair, L1 and L2, and a pinhole), a chopper, and a photodetector (PD). The laser is incident at an angle of 45° onto the sample, which is driven by an electric signal source. The detected signal is analyzed by a lock-in amplifier. The inset presents a photograph of the sample and a cross-sectional image obtained by scanning electron microscopy. (b) Experimentally measured reflection spectrum, with red dots representing experimental data and a solid red line serving as a eye-guide. The slope of the reflection spectrum is presented by a dashed black line. (c) Measured |Δ*R*| for different modulating voltages (blue: results of ±5 V, red: results of ±10 V). (d) Measured |Δ*R*| as a function of modulating voltage at the wavelength of 810 nm. The linear fit to the data points indicates a modulation sensitivity of 0.19 %/V. (e) Measured |Δ*R*| as a function of modulating frequency at a modulating voltage of ±5 V.

In our experiments, the sample was affixed to a custom-designed printed circuit board (PCB). The top and bottom Au electrodes of the sample were connected to the PCB pads using conductive silver glue, as shown in a photograph in Figure 3(a). The cross-section of the sample is shown by a scanning electron microscopic image. The optical setup is sketched in Figure 3(a). To characterize the modulator device, we utilized a linearly polarized laser beam (TUN-TiN, pulse repetition rate of 50 kHz) to excite the sample, which was slightly focused using a lens pair (L1 and L2) to ensure a light spot size smaller than the sample area (2 × 2 mm^2^). The PCB was securely positioned on a home-made holder tilted at an angle 45° relative to the laser beam. The connection between the PCB and an electric modulating source [including an arbitrary signal generator (Agilent 33120A) and a voltage Amplifier (Falco systems WMA-300)] was established via alligator clip wires. Optical signals were captured by a photodetector (Thorlabs DET36A/M) equipped with a lock-in amplifier (Stanford SR830). To measure the reflection spectrum of the modulator, the laser beam was chopped (Thorlabs MC1F30). The optical signals were recorded after the sample at different wavelengths and normalized by the signal measured before the sample. The measured spectrum (Figure 3(b)) demonstrates a reasonable agreement with the simulated result (Figure 2(a)) in terms of resonance shape and position. Furthermore, the reflection slope curve is plotted by a dashed black line, and the modulation amplitude is predicted to peak at 810 and 830 nm.

To assess the electro-optical performance of the modulator, we removed the chopper, and applied square electric voltage to the sample, alternating at the same frequency as the chopper. The resulting |Δ*R*| is shown in Figure 3(c). Results obtained under modulation voltages of ±5 and ±10 V are presented by blue and red data points, respectively. The measured |Δ*R*| exhibits the M-shape similar to the simulated result in Figure 2(d). Two peaks are observed at 810 and 830 nm, consistent with the spectrum slope prediction in Figure 3(a). The maximum |Δ*R*| reaches about 2.3 % at 810 nm under a driving voltage of ±10 V. In Figure 3(d), the dependence of |Δ*R*| on voltages *U* is illustrated, revealing a modulation sensitivity of 0.19 %/V at the wavelength of 810 nm. Furthermore, Figure 3(e) presents the relationship between |Δ*R*| and modulation frequency. Limited by the 50 kHz repetition rate of the laser pulse, the modulation frequency bandwidth is restricted to 25 kHz, and the response curve is flat within this frequency range. Nevertheless, we believe that this is not the upper limit of our modulators, using a laser with a higher repetition rate or continuous output will unleash the modulators’ greater potential.

Table 1 compares our modulator with previous LNOI-based free-space electro-optic modulators, almost of which are based on metasurface designs. Our modulator exhibits the largest modulation amplitude, |Δ*R*|, under driving voltage of similar level. Additionally, our device was fabricated through a one-step metal electrode deposition. Thus, our modulator holds an advantage in terms of easy fabrication compared to other modulators, which are based on metasurfaces designs and require a sophisticated nanostructuring process involving techniques such as focused ion beam milling, electron beam lithography, lift-off, and so on.

**Table 1:** Electro-optic performance of LNOI-based free-space modulators.

Structures	Cut type	Modulation amplitude |Δ*R*| (|Δ*T*|)	*R* (*T*)	Driving voltage (V)	Wavelength range (nm)
LN nanograting [39]	*x*	–	–	±150	633
LN pillars [40]	*x*	0.0054 %	45 %	±5	765–780
LN with gold rings [51]	*z*	–	–	±10	800–900
LN with Al pillars [43]	*z*	–	–	±25	1531–1536
LN with gold nanostripes [52]	*z*	0.8 %	4 %	±10	900–1000
LN with gold nanodisks [41]	*x*	–	–	±2.8	1480–1550
LN with gold grating [42]	*x*	0.25 %	25 %	AC (0 ∼22)	1480–1620
LN with gold nanostripes [44]	*z*	1.26 %	3 %	±10	850–950
**LN thin film (this work)**	***z***	**2.3 %**	**65 %**	**±10**	**800–840**

The values in bold are indicators of our work.

## 3 Discussion

In summary, we have demonstrated an LN thin film electro-optic modulator. Our method leverages the F–P resonance of the LN film clamped between two Au electrode layers. By applying an electric voltage, the refractive index of the LN is changed efficiently, leading to the spectral shift of the F–P resonance and modulation of the reflected light strength at a specific wavelength. A modulation amplitude of 2.3 % is achieved for *p*-polarized light at the wavelength of 810 nm under ±10 V alternating voltage. Comparison with existing metasurface-designed LNOI free-space modulators highlights our device’s superior modulation amplitude and simplified fabrication process. Our results suggest that the planar LN film serves as a viable platform for compact electro-optic modulators in free-space optic manipulation, offering new opportunities for electrically tunable optical components such as beam steering devices, dynamic holograms, and spatial light modulators.
